# Study on dynamic safety distance of multi-storey buildings on the top of loess slope

**DOI:** 10.1038/s41598-022-12282-6

**Published:** 2022-05-17

**Authors:** Lili Wang, Ning Li, Ping Wang

**Affiliations:** 1 Institute of Geotechnical Engineering, Xi’an University of Technology, Xi’an, 710048 China; 2 Key Laboratory of Loess Earthquake Engineering, China Earthquake Administration, 730000 Lanzhou, China; 3 College of Civil Engineering, Lanzhou Jiaotong University, Lanzhou, 730070 Gansu China

**Keywords:** Natural hazards, Civil engineering

## Abstract

In view of the frequent occurrence of strong earthquakes and the actual characteristics of buildings built on the top of slopes in the loess region of Western China, the dynamic response and stability law of loess slope under the building load on the top of the slope are discussed by using the three-dimensional finite element numerical calculation method. Taking the slope safety factor, acceleration amplification coefficient, building permanent displacement and slope sliding range as evaluation indexes, the dynamic safety of buildings on the top of the slope under earthquake is analyzed, and the recommended value of dynamic safety distance between top building and slope shoulder is put forward. The results show that: firstly, affected by the amplification effect of slope elevation, the dynamic response of buildings on the top of slope is stronger than that of buildings under slope. At the same time, affected by the amplification effect of the free surface of the slope, the dynamic response amplitude of the building is negatively correlated with the distance from the slope shoulder. Secondly, the horizontal acceleration ratio between the top building and the under building is the largest at the height of 3–6 m, indicating that the multi-storey building with pile raft foundation is most prone to damage at this height. Thirdly, the dynamic safety distance of multi-storey buildings with pile raft foundation located on the top of loess slope with slope height H ≤ 30 m and slope gradient 25° ≤ α ≤ 70° can be 24 m.

## Introduction

With the promotion of China's "Rural Revitalization" strategy and the construction of new urbanization, urban construction and urbanization in the loess region of western China have developed rapidly. However, there are many gullies and less flat land in this area, so the urban construction land is becoming increasingly less, resulting in a large number of buildings built on loess slopes with different heights and gradient.

**Figure 1:**
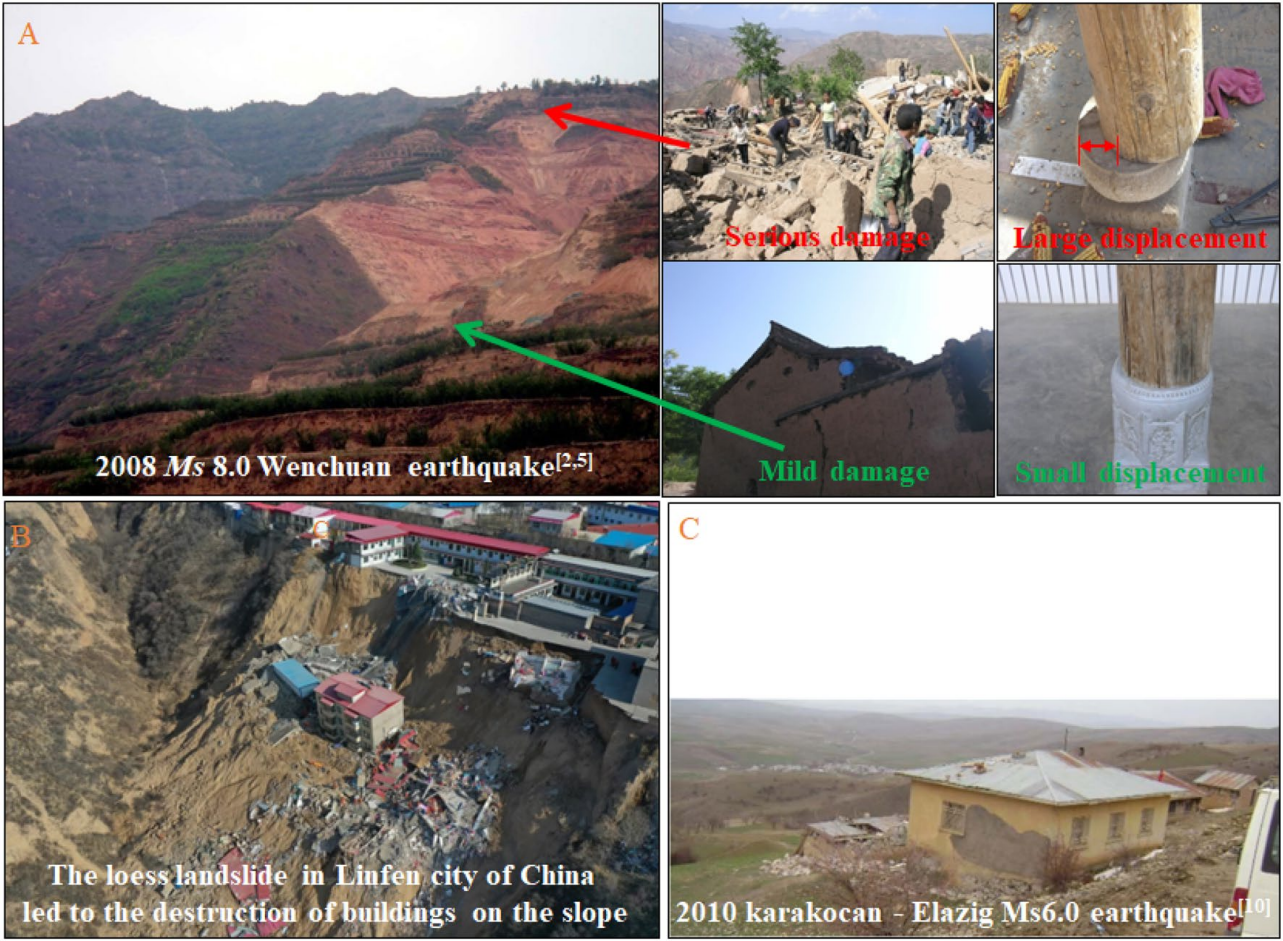
Examples of building failure on top of slope caused by landslide^2,5,10^.

On the one hand, under the influence of seismic elevation amplification effect^1–3^ and thick soil layer amplification effect^4–6^, the dynamic response and damage of buildings on the top of slope will be more serious than those on the horizontal site. At the same time, due to the reflection and refraction of seismic wave on the slope surface, the buildings close to the slope shoulder will be affected by the amplification effect of the surface^7,8^. In the 2008 Wenchuan *Ms*8.0 earthquake, the 2017 China Jiuzhaigou *Ms*7.0 earthquake, the 2010 karakocan—Elazig *Ms*6.0 earthquake^9^ and the 1994 Los Angeles *Ms* 6.6 earthquake, examples of serious damage of buildings on the top of the slope than the horizontal site under the slope were found. On the other hand, when the horizontal distance between the building on the top of the slope and the slope shoulder is too small and the building load on the top of the slope is large, the building load will have an adverse impact on the deformation and stability of the slope, which may lead to the reduction of the stability of the slope^10^, the change of the position and sliding range of the slope, and then lead to the uneven settlement of the building due to the weakening of lateral constraints, cause deformation and overturning of the building. On March 16, 2019, the landslide damage in Linfen City, Shanxi Province of China led to the collapse of buildings on the top of slope, resulting in 7 deaths, 13 injuries. On September 14, 2019, the catastrophic landslide disaster in Tongwei County of China led to the inclined failure of the houses at the top of the slope towards to the slope surface. By analyzing the disasters caused by these kinds of landslides, it is found that the too small slope distance of the building on the slope top is an important reason for casualties. Therefore, it can be seen that the distance between the building and slope shoulder is the key to the degree to which the building on the slope is affected by the elevation amplification effect of the slope, the amplification effect of the free surface and the secondary disasters such as landslide and earthquake subsidence. Therefore, the distance between the building and slope shoulder is an important. Figure 1 shows four examples of building damage caused by seismic wave elevation amplification effect and slope instability.

At present, the commonly used methods for slope stability analysis when considering earthquake action are (a) quasi-static method^11^, (b) dynamic strength reduction method^12^, (c) Newmark method^13–15^, (d) “Pseudo-static approach” + “Strength reduction method”. The essence of quasi-static method is limit equilibrium method. The essence of dynamic strength reduction method is strength reduction method, which needs to be combined with finite element numerical method to calculate the safety factor of slope. The Newmark method takes the yield acceleration as the evaluation index. The yield acceleration refers to the vibration acceleration when the safety factor of the sliding block is 1, which is applicable to the deposit slope. In this paper, the method "d" is used to calculate the safety factor of the slope. This method is essentially a quasi-static method, and the strength reduction method is used to specifically solve the safety factor of the slope. Because the strength reduction method can only analyze the static field, the seismic action is simplified into a horizontal static force, and the gravity field corresponding to the peak strength of ground motion is added in the horizontal direction. This method is to treat the dynamic problem as a static problem, The advantage is that it can comprehensively use the advantages of strength reduction method and quasi-static method, does not need to assume the sliding surface in advance, and has high calculation efficiency. Table 1 summarizes the main principles, advantages and disadvantages of the above four analysis methods.

**Table 1:** Basic principle, advantages and disadvantages of slope dynamic stability analysis method.

Method name	Pseudo-static approach	Dynamic strength reduction method	Newmark	“Pseudo-static approach” + “Strength reduction method”
Basic principles	Based on the limit equilibrium method, the seismic action is simplified as the seismic inertial force acting on the sliding block	The seismic history is divided into equal length steps, and the safety factor of the slope in each step is calculated	When the effective acceleration caused by earthquake is greater than the yield acceleration, the sliding body will slide	Based on the strength reduction method, the seismic action is equivalent to a horizontal gravity field
Disadvantages	The sliding surface needs to be assumed in advance, which can not reflect the dynamic response between the slope and other structures	The calculation time is too long to converge	The yield acceleration is not easy to determine, and this method is only applicable to the slope whose strength does not decrease significantly in earthquake	Compared with the dynamic finite element strength reduction method, the safety factor calculated by this method is an approximate solution,
Advantages	The concept is clear, the calculation workload is small, and the parameters are easy to determine	The variation curve of safety factor with ground motion time history can be obtained	Considering the deformation safety of slope, it is suitable for deposit slope or rock slope	Combining the advantages of strength reduction method and quasi-static method, the calculation time is shorter than that of dynamic strength reduction method, and there is no need to assume the sliding surface in advance

There are two main indexes for evaluating the stability of soil slope under earthquake: seismic permanent deformation and stability safety factor^16,17^. Critical acceleration, critical displacement and plastic strain zone penetration rate^18–20^ are often used as specific evaluation indexes in numerical analysis to evaluate slope stability. Due to the topographic reasons of the slope, the seismic wave elevation amplification effect from bottom to top and the free surface amplification effect from inside to outside are produced. Therefore, the dynamic amplification factor is also an important evaluation index to judge the dynamic stability of the slope and the building on the top of slope. When the slope has permanent deformation but no sliding failure and the safety factor is still within the safe range, the buildings on the slope may be in the critical state of instability due to the settlement and inclination. Therefore, due to the existence of buildings on the slope, more strict requirements should be put forward for the deformation of the slope. When studying the slope and buildings on the slope as a system, the stability evaluation of the slope should take into account the deformation safety of the building.Due to the existence of buildings on the top of slope, more strict requirements are put forward for the deformation of the slope. Therefore, when the buildings and the slope are studied as a system, the stability evaluation of the slope should take into account the deformation safety of the buildings. In fact, taking the Wenchuan earthquake as the node, the seismic fortification capacity and level of Chinese buildings have been greatly improved. By summarizing the damages of buildings on the top of slope caused by strong earthquakes and landslides^21–23^, The damage of buildings is mainly deformation damage and overall collapse with the slope. Therefore, the permanent deformation of the building, the potential sliding range of the slope and the dynamic amplification factor of the building should be important indicators to evaluate the safety of the buildings on the slope.

In view of the above analysis, based on the previous research on the dynamic response law of high-steep loess slope under building load^24,25^, the distribution characteristics of slope buildings in loess area are further investigated and counted, and three-dimensional models considering the influencing factors of slope height, slope gradient and the distance between top buildings and slope shoulder are established. The safety factor, acceleration and displacement response law of loess slope under earthquake are discussed. This manuscript attempts to take the acceleration and displacement attenuation law of slope top, the permanent displacement of the building, the slope safety factor and the slope sliding range as the evaluation indexes to evaluate the safety of multi-storey buildings on the top of slope. The research results can provide a scientific basis for the site selection of buildings on the top of slope in loess area, and provide a new criterion for the instability of soil slope under building load and seismic load.

## Distribution characteristics of buildings on top of slopes in loess region of China

**Figure 2:**
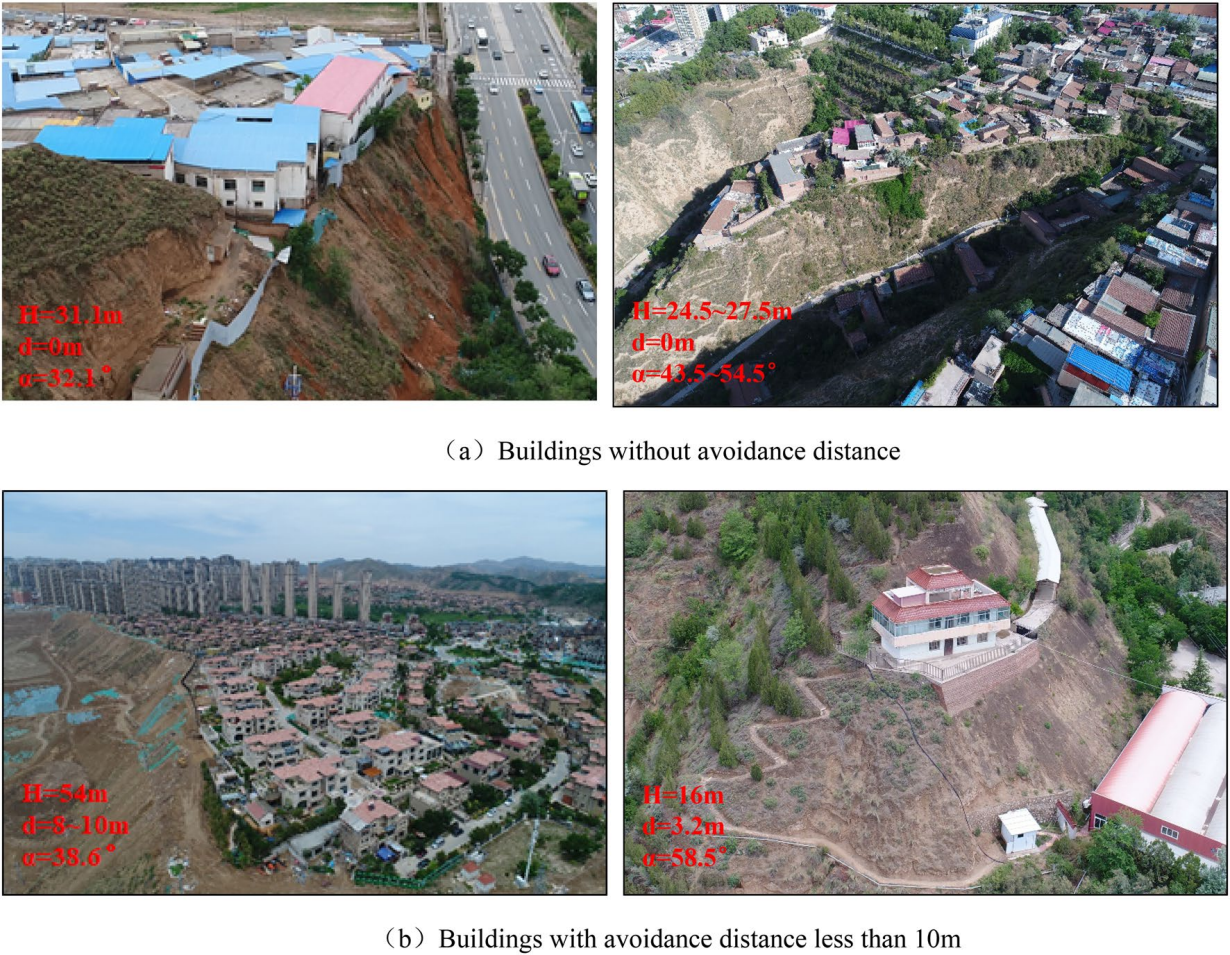
Typical buildings on the top of loess slope in the west of China.

The research group focused on the investigation and statistics of rural houses and urban buildings built on slopes in three key provinces in the loess region of Western China, Gansu Province, Shanxi Province and Shaanxi Province. According to the investigation and statistical analysis results, the buildings on the top of slope can be divided into two categories: ① The buildings are close to the slope shoulder without avoidance distance. Most of the buildings are single-layer or two-layer agricultural houses. The building foundation is mainly rubble foundation or expanded foundation, and the building structure is mainly soil wood structure, brick wood structure and brick concrete structure. ② The avoidance distance of buildings is random, most of which are within 10 m. The buildings are mainly multi-storey masonry, and the building foundation structure is mainly pile raft foundation. The gradient and height of the building slope meet the following characteristics: the gradient range of slope: α = 25°–70°, the vertical height of single grade slope from slope toe to slope shoulder: H = 15 m ~ 60 m, and most single-stage slopes are concentrated in the range of 15 m ~ 30 m. Figure 2 shows some typical buildings on the top of loess slope in the west of China.

## Model settings

Based on the above survey results and combined with the research results of site amplification effect^26^: the amplification effect of loess site is more obvious when the thickness of soil layer is 15 m < H < 30 m. Therefore, three-dimensional numerical models considering the influencing factors of slope height, slope gradient and the distance between top buildings and slope shoulder are established by using Midas GTS software. The specific geometric dimensions of the slope model are shown in Table 2. In Table 2, the distance between building and slope shoulder is from 5 to 35 m, the reason is: Firstly, based on the research results of "1. Distribution characteristics of buildings on top of slopes in loss region of China", the slope distance of buildings in loess area is within 10 m, so 5 m and 10 m are set. Secondly, in order to explore the attenuation relationship between the dynamic response of buildings and the distance between top building and slope shoulder, two cases of 20 m and 35 m are set up. Due to the lack of construction land in the loess area, if the distance is too larger, it will no longer meet the original intention of building on the top of the slope. Therefore, set the distance between building and slope shoulder as 5 m, 10 m, 20 m and 35 m.

**Table 2:** The specific geometric dimensions of the slope model.

Slope height (H/m)	Slope gradient (α/°)	Distance between building and slope shoulder (d/m)
15 m, 30 m	25°,45°,70°	5 m, 10 m, 20 m, 35 m

The building size on the slope is long * wide * height = 30 m * 12 m * 24 m, simulating an 8-storey building of 24 m, the building foundation adopts pile foundation, and the foundation spacing is 4 m × 5 m, 12 m long pile, the section size is 1 m × 1 m. As combined with the code for design of concrete structures (Chinese GB50010-2010), the model material parameters are shown in Table 3.

**Table 3:** Material parameters.

	E/Mpa	μ	γ/kN.m^-3^	c/kPa	φ/°
Slope soil	50	0.35	16.6	82.5	23.4
Pile	31,000	0.2	23	–	–
Buileding	5000	0.2	21	–	–

**Figure 3:**
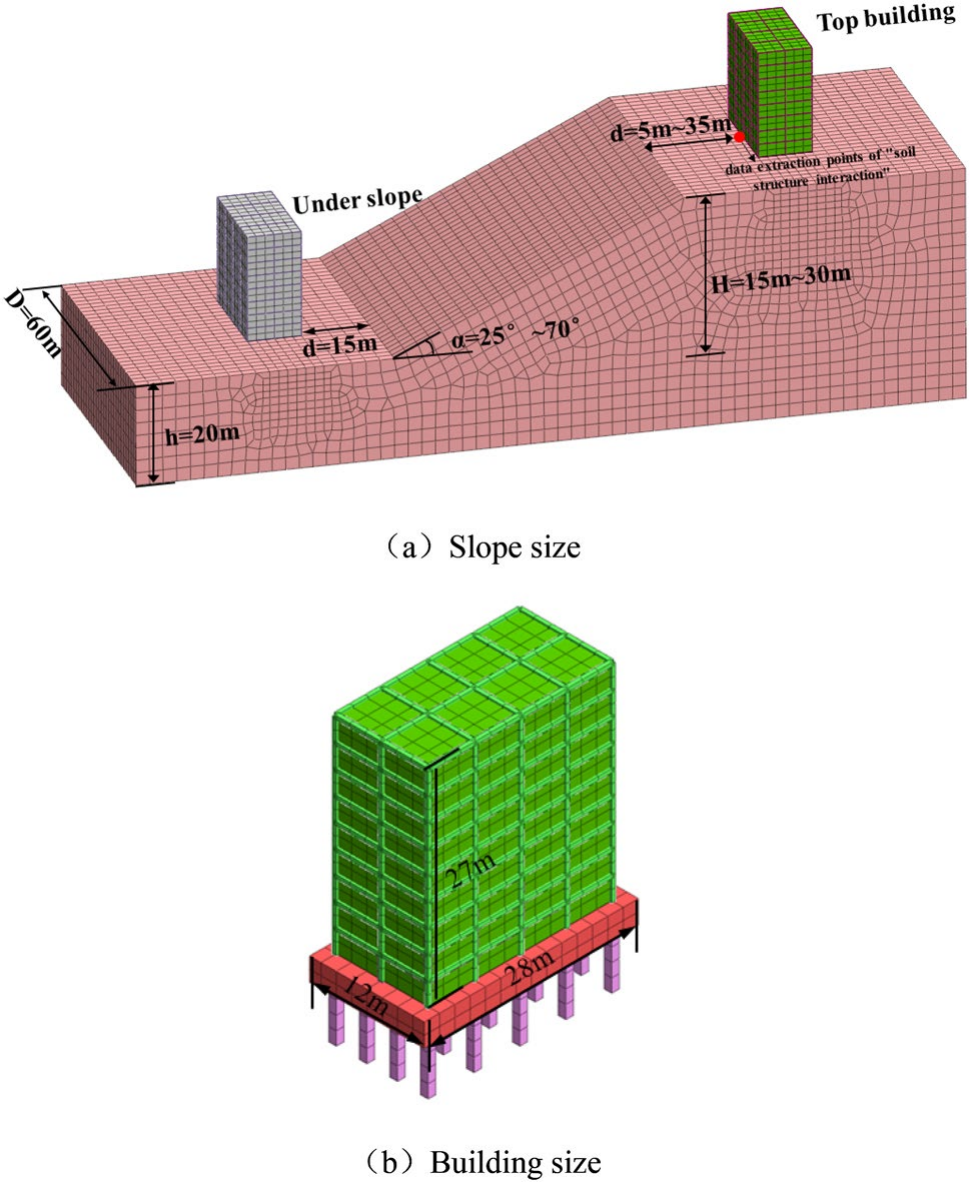
Model size.

Figure 3 shows the geometry of the numerical model. In this study, the distance between the building on the top of the slope and the slope shoulder is defined as **d**, the height of the slope is represented by H. In this study, the distance of **d** is set with four variables: d = 5 m, d = 10 m, d = 20 m and d = 35 m, and the height of the slope is set with two variables: H = 15 m and H = 30 m.

**Figure 4:**
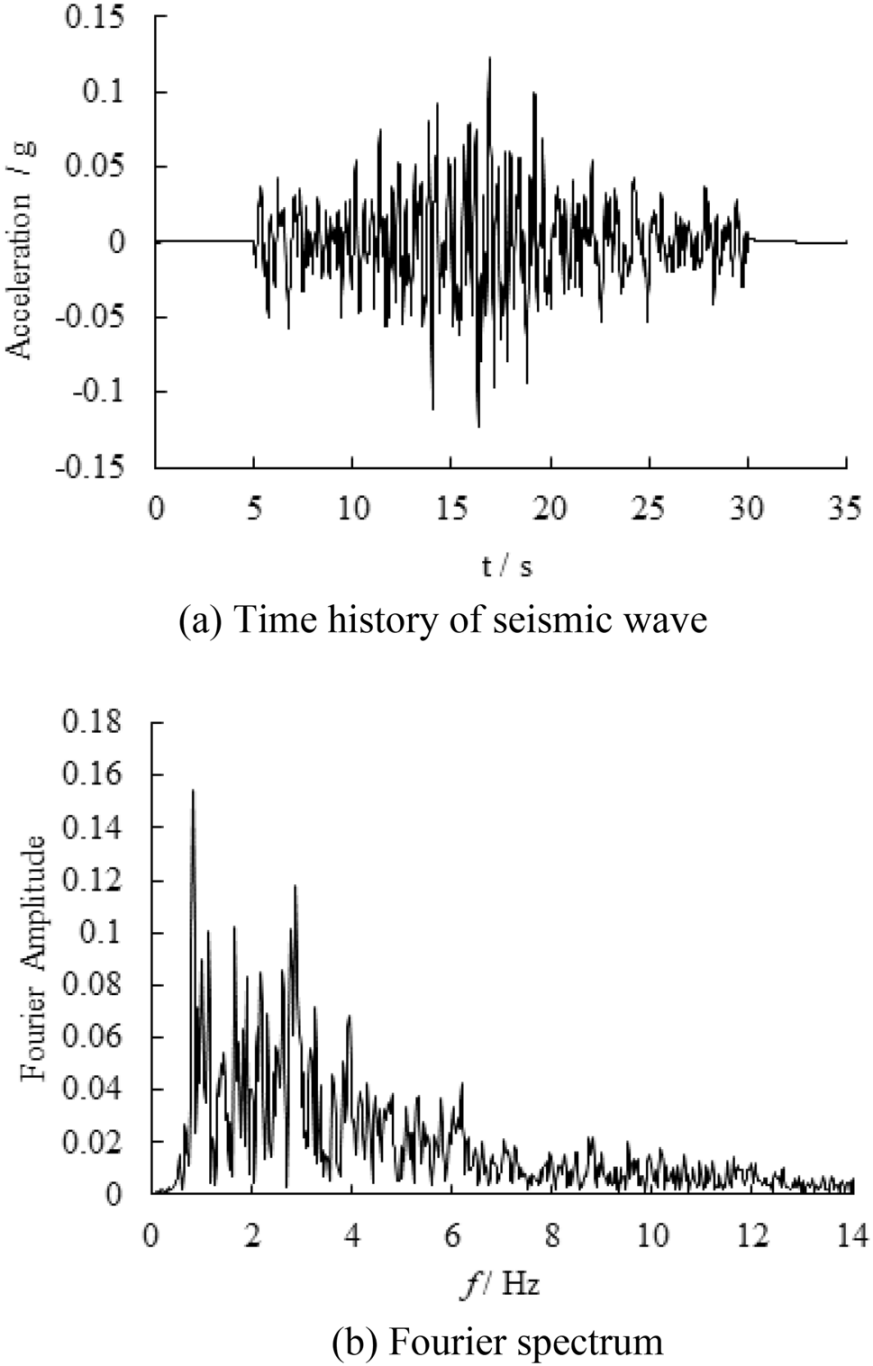
The time history of seismic wave and fourier spectrum.

In this manuscript, the nonlinear time history analysis is carried out. The boundary of the models are viscoelastic boundary, which is used to simulate the infinite region in seismic analysis. The seismic wave adopts the far-field wave collected by Wenchuan earthquake in Tangyu Town, Shaanxi Province of China. The seismic peak acceleration is 0.12 g, and its Fourier spectrum is bimodal. The predominant frequency of the first main peak is 0.83 Hz, and the predominant frequency of the second main peak is 2.87 Hz. The seismic wave time history and Fourier spectrum are shown in Fig. 4. Filtering and baseline correction are carried out before seismic wave input, and the filtering frequency band is 0.1–25 Hz.

## Influence analysis of elevation amplification effect on buildings

**Figure 5:**
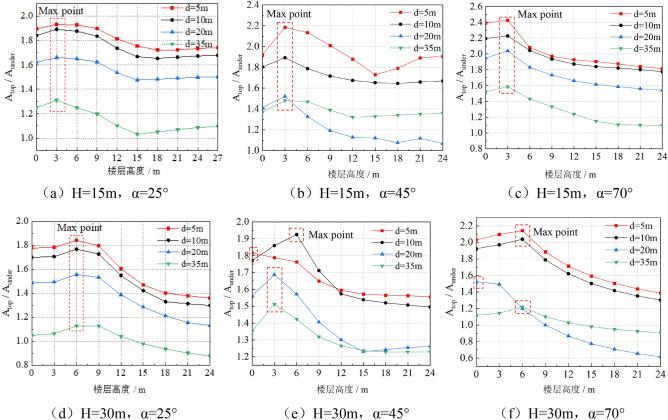
the relative variation law of the acceleration ratio of the top building and the under building along the building height.

In order to analyze the influence of the elevation amplification effect on earthquake resistant behavior of building, the relative variation law of the horizontal peak acceleration ratio of the top building and the under building along the building height is summarized as shown in Fig. 5. In Fig. 5, the ordinate A_top_/A_under_ is the horizontal peak acceleration ratio of the top building to the under building, The abscissa h in the Fig. 5 is the vertical height of the measuring point along the building height direction.

According to the analysis of Fig. 5, when H = 15 m, the maximum horizontal peak acceleration ratio of buildings on the top of slope to buildings under the slope is located at the point of h = 3 m, that is, the top of the first floor and the bottom of the second floor. When H = 30 m, the position of the maximum horizontal peak acceleration ratio of the building on the top of the slope and the building under the slope is different from the model of H = 15 m, which is not fixed, but relatively concentrated at the position of 3 m ≤ h ≤ 6 m.

**Figure 6:**
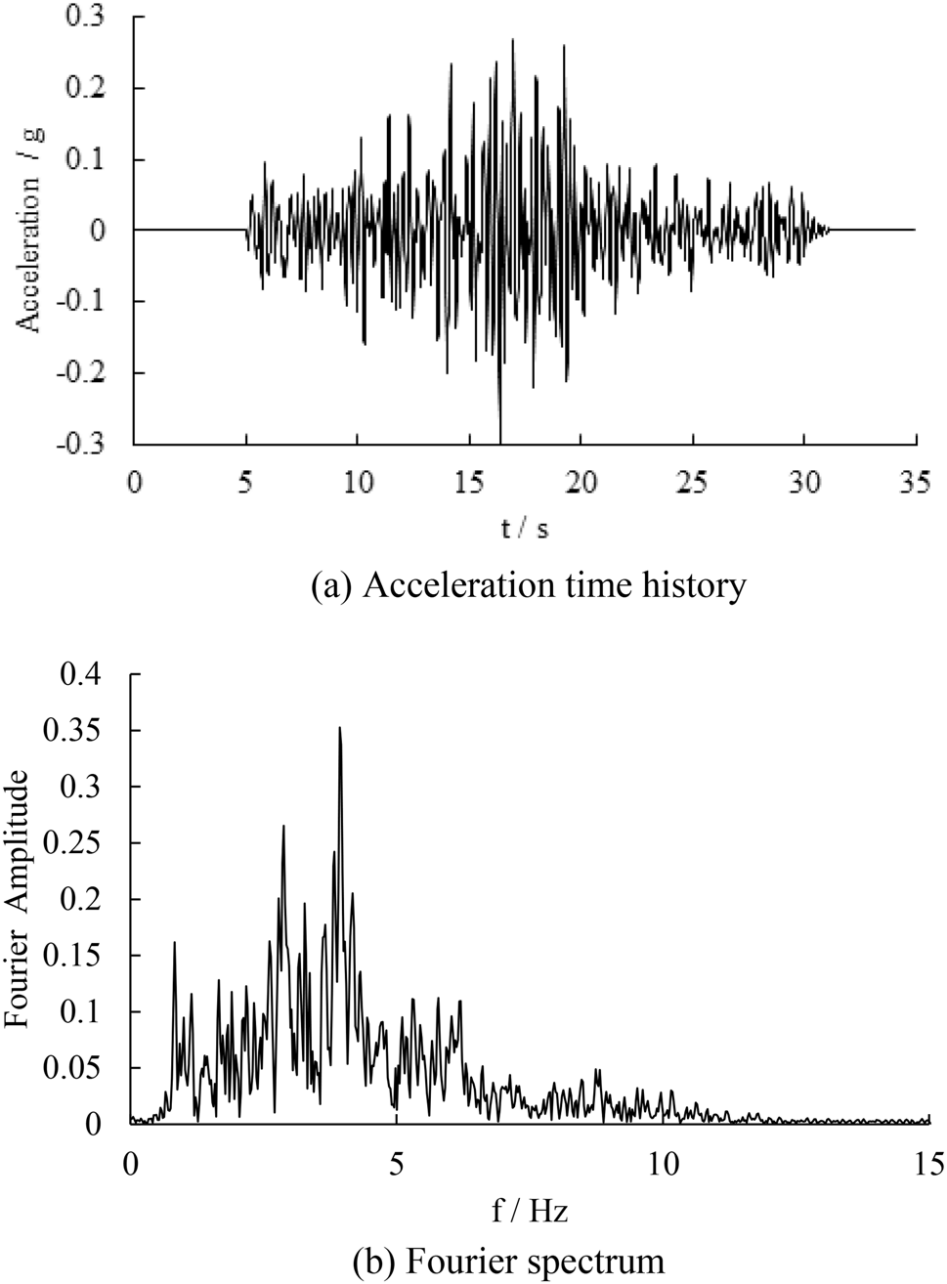
Acceleration time history and Fourier spectrum of top buildings at h = 3 m point of H = 15 m, α = 25°, d = 5 m model.

In order to analyze the reason why the horizontal acceleration is amplified within the height range of 0 ~ 6 m of the building, taking the model of H = 15 m, α = 25°, d = 5 m as an example, the horizontal acceleration time history of the top building at h = 3 m is extracted, and the corresponding Fourier spectrum is analyzed, as shown in Fig. 6.

**Figure 7:**
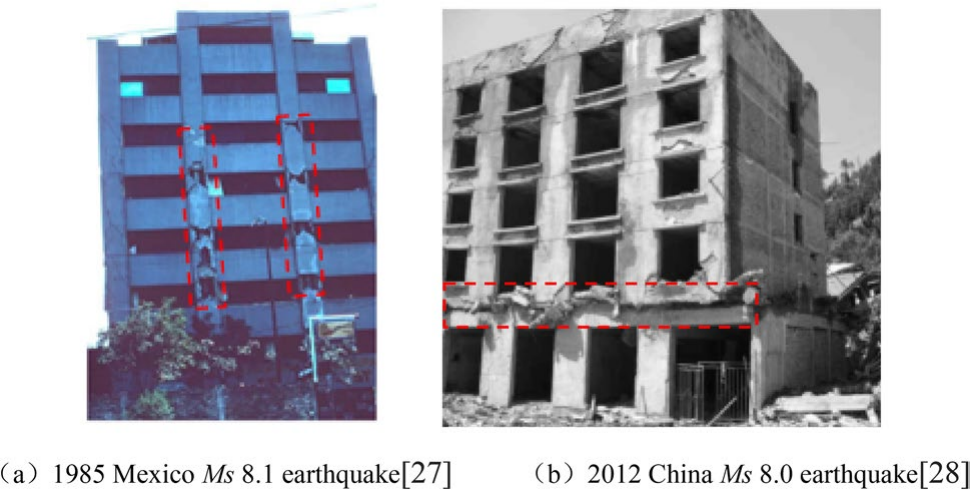
Examples of earthquake damage in the middle of multi-storey buildings.

As can be seen from Fig. 6, the model with H = 15 m, the predominant frequency of Fourier spectrum at h = 3 m is 3.93 Hz, which is very close to the first-order main mode frequency of the model of 3.29 Hz, therefore, the seismic amplification effect is obvious at h = 3 m. Similarly, for the model with H = 30 m, the maximum value of A_top_/A_under_ is mostly located at h = 3 m ~ 6 m, which is also related to the fact that the natural frequency of those heights is close to the predominant frequency of the input seismic wave. Some examples of the destruction of multistory buildings from the middle parts was found in 1985 Mexico *Ms* 8.1 earthquake^27^ and 2012 China *Ms* 8.0 earthquake^28^, as shown in Fig. 7. The most likely reason is that the natural vibration period of the middle part of the building is close to the superior frequency of the input seismic wave, resulting in the resonance failure of the middle layer.

**Figure 8:**
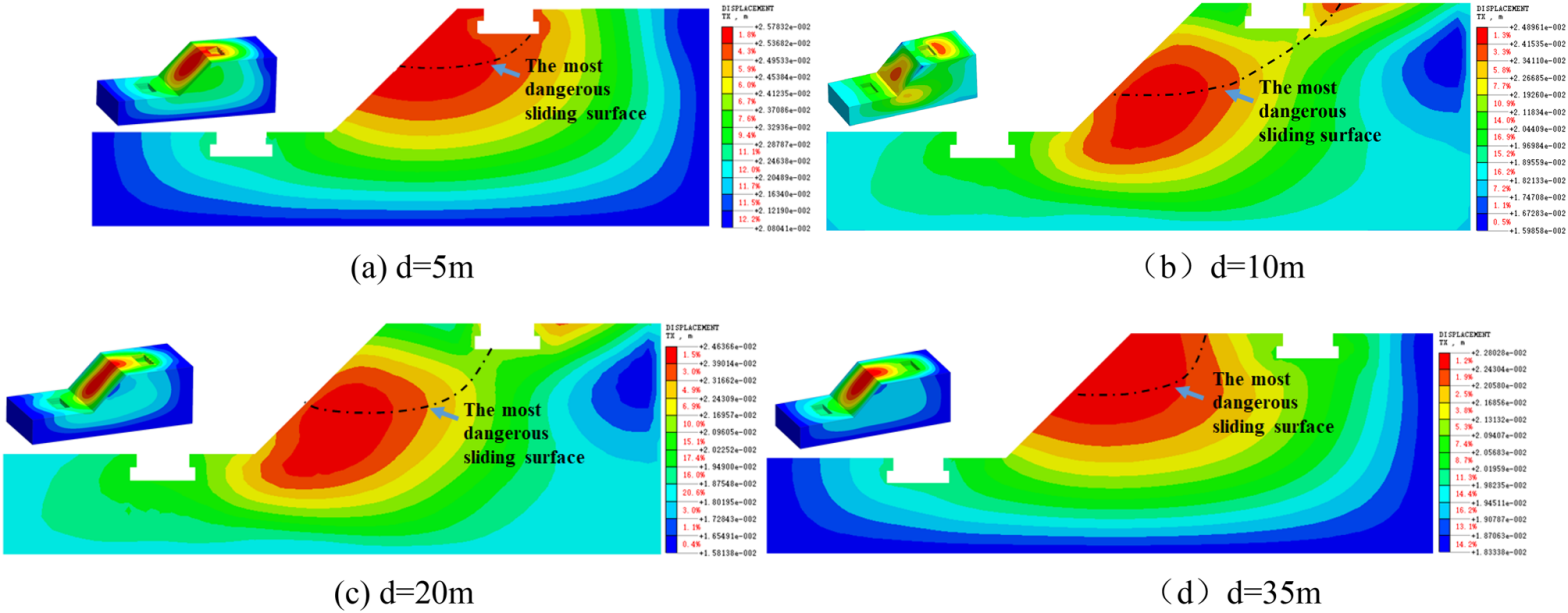
Horizontal displacement nephogram of models with H = 30 m, α = 45°.

It can also be seen from Fig. 5 that the amplification effect is negatively correlated with the distance of **d**, that is, the smaller of the **d**, the higher the value of A_top_/A_under_ will. But at H = 30 m, α = 45°, when the distance of the d is 10 m, the value of A_top_/A_under_ is greater than d is 5 m. In order to analyze the reason, the horizontal displacement nephogram of H = 30 m, α = 45°models is compared and analyzed, as shown in Fig. 8. Figure 8 shows the most dangerous sliding surface of the slope with a black dotted line according to the distribution and change law of the displacement nephogram.

As can be seen from Fig. 8, When d = 5 m, only part of the top building is located within the most dangerous sliding area of the slope, while when d = 10 m, all parts of the building are located within the most dangerous sliding area of the slope. When d = 20 m and 35 m, with the increase of the distance of **d**, the buildings gradually move away from the most dangerous sliding surface of the slope. Therefore, the model with height H = 30 m and the gradient α = 45°, when the distance between top building and slope shoulder is 5 m, the top building will have the highest risk of sliding failure with the slope.Therefore, in Fig. 5e, the acceleration amplification effect of the curve d = 10 m is the most obvious. so when H = 30 m, α = 45°,d = 10 m, the site amplification effect is the most obvious.

The displacement 5 s after the end of earthquake is defined as the permanent displacement. The variation of the permanent displacement of the top floor of the building with the distance of **d** is analyzed, as shown in Fig. 8.

**Figure 9:**
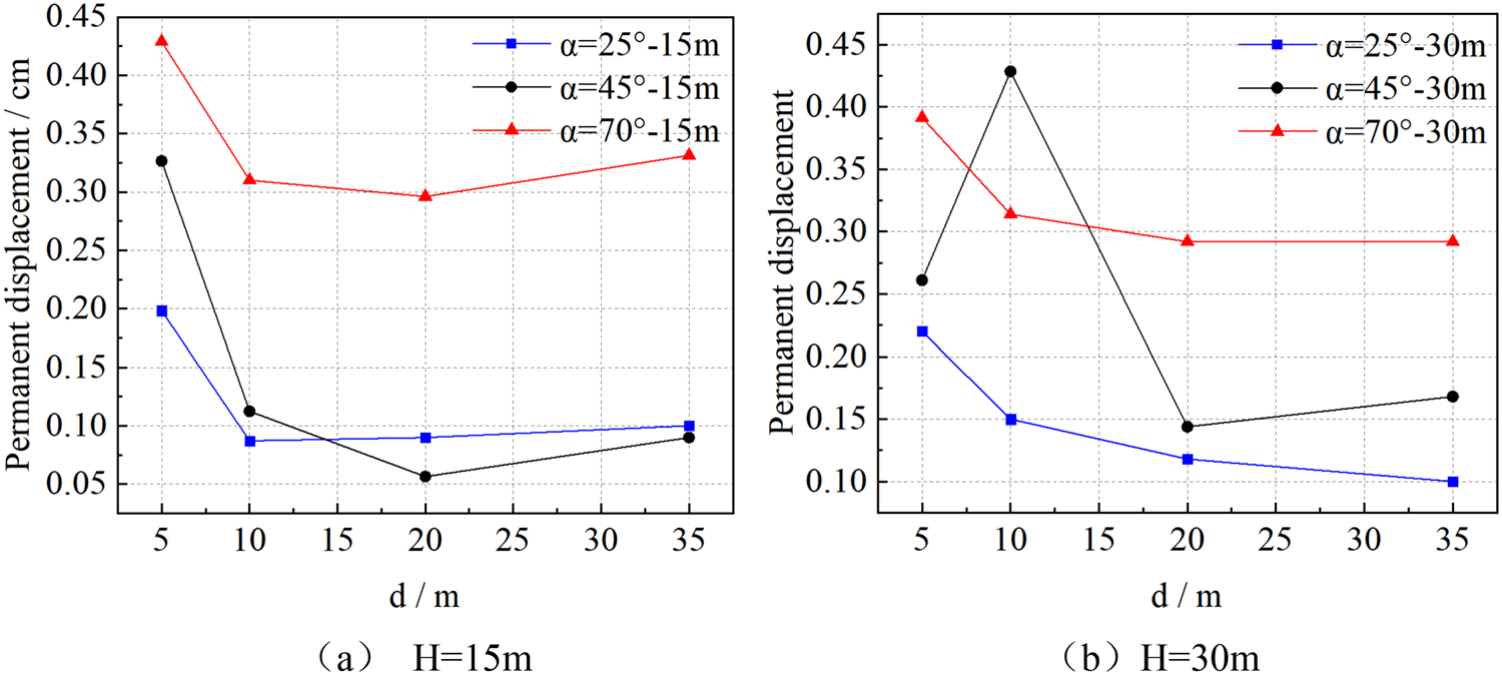
Variation curve of permanent displacement of top building with distance of **d**.

Figure 9 shows that the change of the distance between the top building and slope shoulder has an important impact on the permanent displacement of the building. The overall change trend is that the permanent displacement of the building on the top of the slope decreases with the increase of the distance of **d.** The specific discussion is as follows: when H = 15 m, the building on the top of the slope takes **d** = 20 m as the inflection point, before the inflection point, the permanent displacement of the building increases due to the amplification effect of the free surface of seismic waves, after the inflection point, the permanent displacement of the building increases due to the boundary effect of the model. When H = 30 m, the permanent displacement decreases slowly after d ≥ 20 m. Therefore, when the permanent deformation of the building is used as the evaluation index to measure the damage degree of the building, **d** = 20 m can be used as the dynamic safety distance of the building on the slope. In Fig. 8b, there are abnormal points of change, i.e. at the point of H = 30 m, α = 45°, **d** = 10 m, the permanent displacement of the building increases abnormally, which is consistent with the sudden increase of the ratio of the peak value of horizontal acceleration of buildings on and below the slope shown in Fig. 5e, which also verifies the rationality of the analysis result of elevation amplification effect.

## Influence of distance between top building and slope shoulder on dynamic response law of slope

**Figure 10:**
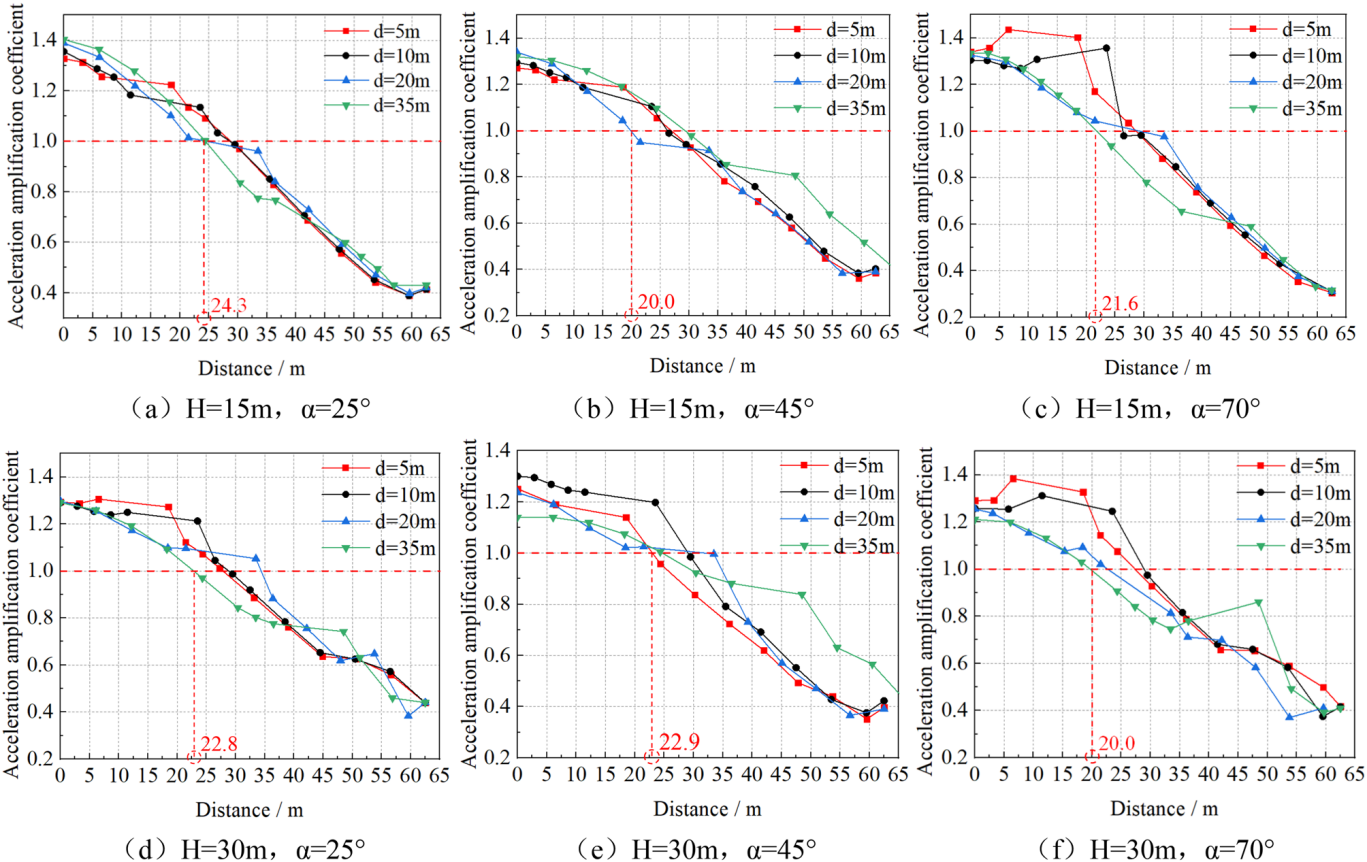
Acceleration amplification coefficient at the top of slope VS distance from slope shoulder.

The acceleration amplification coefficient is defined as the ratio of the horizontal acceleration of each measuring point on the slope top to the horizontal acceleration of the slope toe. Along the central axis of the slope, extract the acceleration of each measuring point with different distance from the slope shoulder on the top slope, and analyze the variation law of the acceleration amplification coefficient with the distance between each measuring point and the slope shoulder, as shown in Fig. 10. The abscissa of Distance is the horizontal distance between the measuring point on the top of slope and the slope shoulder.

According to the analysis of Fig. 10, firstly, according to the horizontal coordinate value of the intersection of the line with acceleration amplification coefficient is1 and other acceleration amplification coefficient curves, the minimum dynamic safety distance of the building on the top of slope can be taken as 20–24.3 m. Secondly, under the influence of soil -structure interaction, the acceleration of soil near the building foundation increases sharply, and this sudden increase effect is more obvious with the increase of slope gradient and height. Thirdly, the acceleration amplification coefficient at the top of the slope is reduced to about 0.4 times at the right boundary, which is due to the essence of the viscoelastic boundary, that is, damping plays an absorbing role at the boundary. Try to expand the boundary length by 100 m, and the acceleration near the boundary is still small. Therefore, the specific value of acceleration at the boundary is not discussed here, and only the overall change trend is discussed.

**Figure 11:**
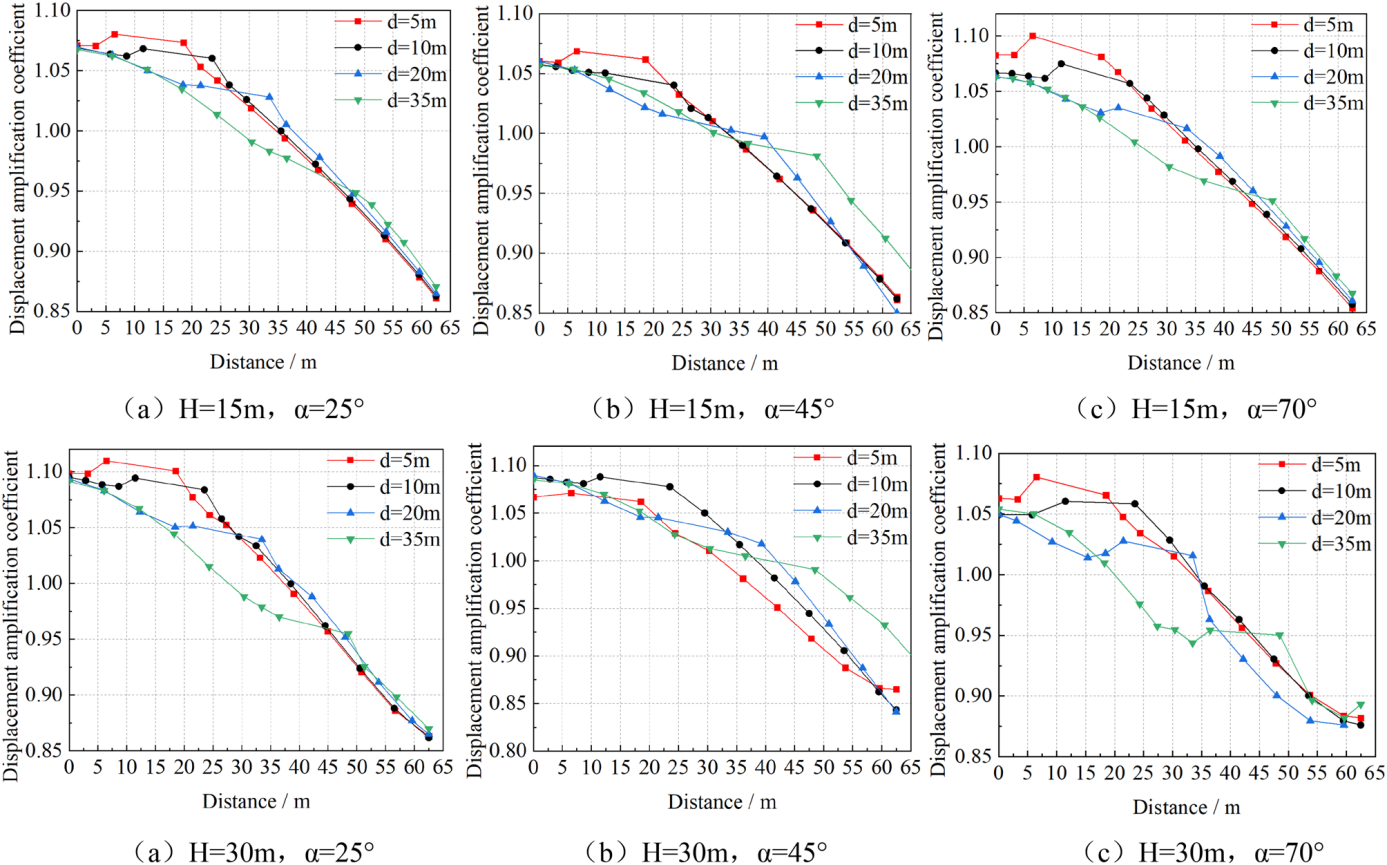
Displacement amplification coefficient of top slope VS distance from slope shoulder.

Similarly, the displacement curve of each measuring point on the top of slope with the distance from the slope shoulder is statistically analyzed, as shown in Fig. 11. The displacement amplification coefficient is defined as the ratio of the horizontal displacement of each measuring point on the slope top to the horizontal displacement of the slope toe.

As can be seen from Fig. 11 the variation law of displacement is similar to that of acceleration: with the increase of the distance, the displacement amplification coefficient gradually decreases, and the displacement amplification coefficient suddenly increases at the junction of building foundation and soil. The smaller the distance, the more obvious the displacement amplification factor of the soil near the building foundation increases. The magnification of displacement is less than that of acceleration.

In the above analysis, it is concluded that the amplification effect of acceleration and displacement increases significantly at the junction of building foundation and soil. Further statistical analysis is carried out on 24 models with different slope height, gradient and distance of d. It is found that the influence range of soil amplification effect near the foundation caused by "soil-structure interaction" is within 3 m-6 m, in order to avoid the influence of this interaction between buildings, the distance between multi-storey frame buildings shall be greater than 12 m.

**Figure 12:**
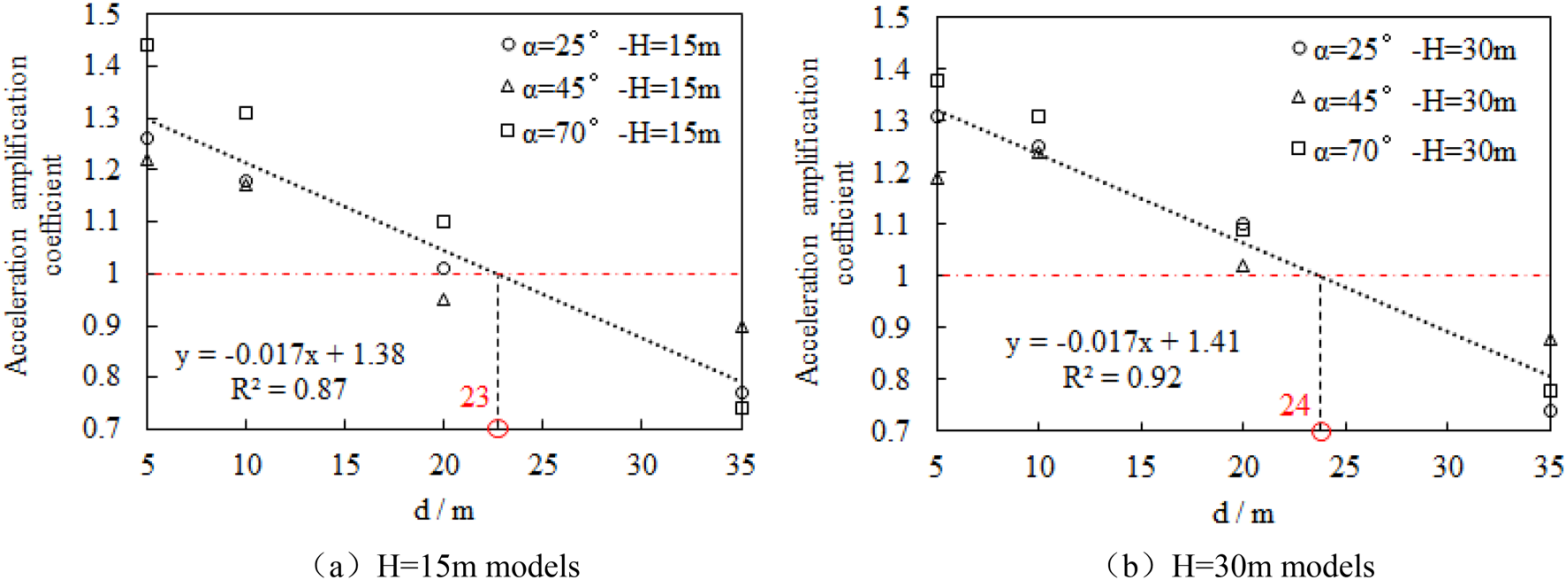
Variation curve of soil acceleration amplification coefficient at building foundation on top slope with building distance of **d**.

The variation law of acceleration amplification coefficient at the joint of building foundation and slope soil with the distance of **d** is statistically analyzed, as shown in Fig. 12

.

According to the analysis of Fig. 12, the acceleration amplification factor of the soil at the building foundation gradually decreases with the decrease of the distance of **d**, which shows that the building dynamic amplification characteristics caused by the amplification effect of the free surface of the slope decrease with the increase of the distance of **d**. The scatter plots in Fig. 12 are linearly fitted. The intersection of the fitted trend line and the horizontal line with the acceleration amplification coefficient of 1 time is the point where the building on the top of slope is no longer affected by the amplification effect of the free surface of the slope. Therefore, d = 23 m and d = 24 m should be the dynamic safety distance points of the building of H = 15 m and H = 30 m slope respectively.

## Influence of the distance between building and slope shoulder on slope safety

**Figure 13:**
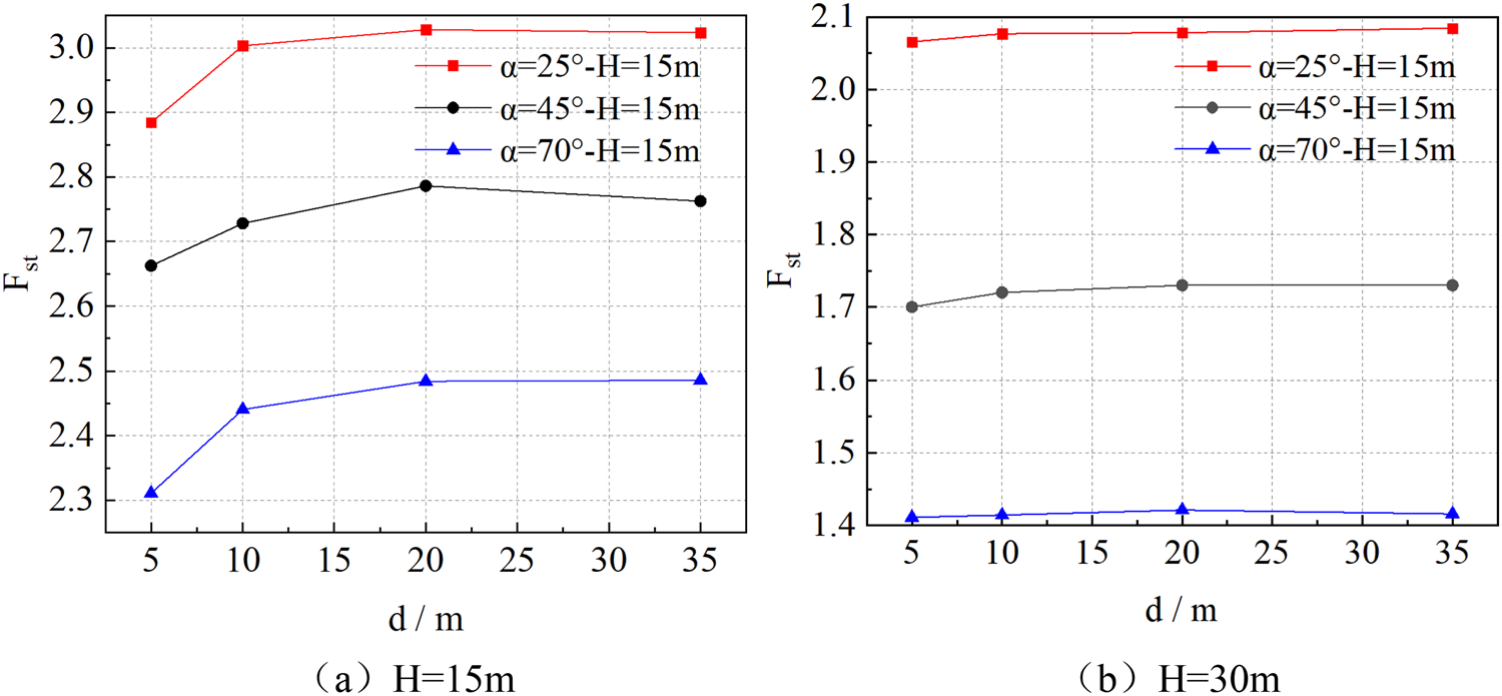
The safety of slope VS **d**.

The safety factor of the slope is calculated by the combination of strength reduction method and quasi-static method. The variation curve of the safety factor of each slope model with the distance of **d** is shown in Fig. 13.

As shown in Fig. 13, firstly, when H = 15 m, the safety factor of the slope increases gradually with the increase of the distance of **d**. when the **d** increases to 20 m, the safety factor tends to not change again. When H = 30 m, similar to the slope with H = 15 m, when **d** increases to 20 m, the safety factor basically does not change. The difference is that the safety factor of slope is less affected by the change of distance of **d**. Secondly, with the increase of slope gradient, the safety factor of slope decreases linearly, and the slope models with two different heights are in line with this change characteristic.

**Figure 14:**
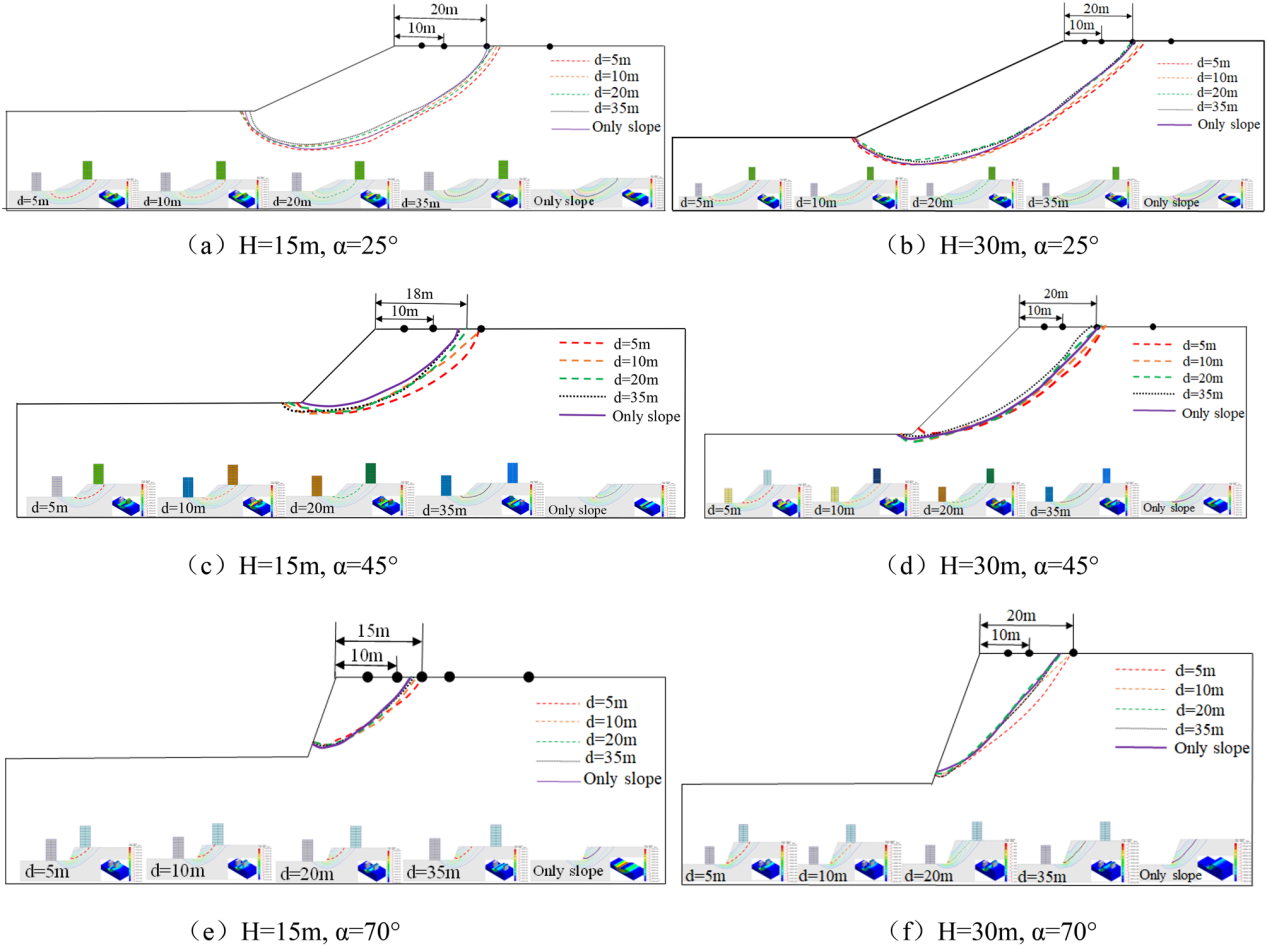
Summary drawing of the most dangerous sliding surface of each model.

According to the isoline change trend of the maximum shear strain nephogram, the most dangerous sliding surface of each slope model is summarized as shown in Fig. 14.

As can be seen from Fig. 14: ① The most dangerous sliding surface of loess slope is related to the distance of **d**. The smaller the distance of **d**, the larger the instability range of the slope. ② α = 25°, i.e. when the slope gradient is gentle, the sliding surface of the model with the building distance d = 20 m tends to be consistent with the sliding surface without the building model, which indicates that the slope effect basically disappears and the buildings on the slope top tend to be safe after the building distance **d** > 20 m. ③ When α = 45°, H = 15 m, the most dangerous sliding surface of the pure slope is 15 m away from the slope shoulder, while the most dangerous sliding surface of the **d** = 20 m model is 18 m away from the slope shoulder, indicating that the building on the slope top will not slide with the sliding failure of the slope after the building distance **d** > 20 m. When α = 45°, H = 30 m, the most dangerous sliding surface of the slope model without building is 20 m away from the slope shoulder, and the most dangerous sliding surface of the **d** = 20 model is close to the pure slope model, indicating that the slope effect of the building basically disappears after **d** > 20 m, and the building on the slope top tends to be safe. ④ When α = 70°, H = 15 m, the sliding range of the slope is between 10 and 15 m. Therefore, when the building distance of **d** > 15 m, the building will not slide with the instability of the slope. When α = 70°, H = 30 m, the sliding range of the slope is between 18 and 20 m, and after building distance of **d** > 20, the most dangerous sliding surface of the model is close to the model without building, indicating that after **d** > 20 m, the slope effect basically disappears and the buildings on the slope top tend to be safe.

To sum up, from the perspective of avoiding the most dangerous sliding surface of the slope, the loess slope with α = 25°, the dynamic safety distance of building on the top of slope is 20 m; For the loess slope with α = 45°, when H = 15 m, the dynamic safety distance of building on the top of slope is 18 m; when H = 30 m, the dynamic safety distance of building on the top of slope is 20 m. For α = 70° loess slope, when H = 15 m, the dynamic safety distance of building on the top of slope is 15 m, and when H = 30 m, the dynamic safety distance of building on the top of slope is 20 m.

## Dynamic safety distance of multi-storey frame structure on the top of loess slope

Based on the impact analysis of elevation amplification effect on top buildings, the impact analysis of building distance of **d** on slope dynamic response law and the impact analysis of building distance of **d** on slope safety, respectively, Taking the attenuation law of the acceleration amplification factor of the soil near the building foundation as the index, and according to the change of the safety factor and sliding range of the slope, the dynamic safety distances d_1_, d_2_ and d_3_ of multi-storey frame building of pile raft foundation on slope are summarized in Table 3.

Considering the influence of the slope distance of the top building on the slope stability, taking the slope safety factor as the evaluation index, the dynamic safety distance of the multi-storey frame building with pile raft foundation on the top of slope is defined as d_1_. Considering the relationship between the permanent deformation of the top building and the distance of **d**, taking the displacement of the building 5 s after the earthquake as the evaluation index, the minimum safe distance between the multi-storey frame building with pile raft foundation on the top of slope is defined as d_2._ Considering that the "soil structure" interaction is affected by the amplification effect of free surface of seismic wave, taking the acceleration amplification coefficient of soil near the building foundation on the top of slope as the evaluation index, the minimum safe distance between the multi-storey frame building with pile raft foundation on the top of slope is defined as d_3._ Considering the position relationship between the sliding surface of the slope and the top building, taking the sliding range of the slope as the evaluation index, the minimum safe distance between the multi-storey frame building with pile raft foundation on the top of slope is defined as d_4._ Finally, the dynamic safety distances d_1_, d_2_, d_3_ and d_4_ of the multi-storey frame building with pile raft foundation on the slope are summarized, as shown in Table 4

**Table 4:** Dynamic safety distance of multi-storey frame building with pile raft foundation on the top of slope.

α/°	H/m	d_1_/m	d_2_/m	d_3_/m	d_4_/m
25°	15 m	20	20	23	20
	30 m	20	20	24	20
45°	15 m	20	20	23	18
	30 m	20	20	24	20
70°	15 m	20	20	23	15
	30 m	20	20	24	20

.

When the stability of the slope and the permanent deformation safety of the top buildings are comprehensively considered, the dynamic safety distance of the buildings on the top of slope can be 20 m. Considering the effect of free surface amplification of seismic wave on the "soil structure" interaction of soil near the building foundation, the dynamic safety distance of the buildings on the top of slope can be 24 m. When the slope stability is low and the landslide risk is high, the dynamic safety distance of buildings on the top of slope should be d_4_ from the perspective of avoiding common sliding of buildings with the slope. Influencing factors α and H are fitted with the dependent variable d_3_ to obtain the relationship as shown in (2), R^2^ = 0.70.

1 $${\text{d}}_{{4}} = 0.{\text{156H}} - 0.0{55}\alpha + {17}.{93}$$

## Discussion

The distance between the top building and slope shoulder proposed in this paper is based on the conclusion obtained by considering four evaluation indexes: the sliding range of the slope, the safety factor of the slope, the permanent deformation of the building and the acceleration amplification factor. Among the four evaluation indexes, the distance obtained by taking the sliding range of the slope as the evaluation index is the smallest, and the distance obtained by taking the permanent deformation of the building as the evaluation index is the largest. According to different fortification requirements, when the buildings on the slope top are required not to produce serious deformation and damage under the action of earthquake, the slope safety distance of the buildings on the top of slope should be given with the permanent displacement of the buildings as the evaluation index. When the construction land is very limited, starting from the fortification concept of "no collapse in a large earthquake", the dynamic safety distance can be determined according to the most dangerous sliding range of the slope.

## Conclusion

This manuscript discusses the dynamic stability of loess slope under the building load on the top of the slope, discusses the dynamic response law of buildings on the top of slope under the amplification effect of elevation and free surface, and puts forward the dynamic safety distance of multi-storey buildings on the top of slope. The main conclusions are as follows.

1. When the building is located on the top of the slope, the damage degree of "soil structure" interaction to the building and slope is negatively correlated with the distance between the building and slope shoulder.
2. Taking the slope safety factor, acceleration amplification factor, building permanent displacement and slope sliding range as evaluation indexes, the slope safety of buildings on the top of loess slope under seismic load is analyzed, and the dynamic safety distance value of multi-storey buildings with pile raft foundation on is put forward. Without considering the influence of slope height and gradient, the dynamic safety distance of multi-storey buildings with pile raft foundation located on the top of loess slope with slope height H ≤ 30 m and slope gradient 25° ≤ *α* ≤ 70°can be 24 m.
3. The ratio of the peak value of the horizontal acceleration of the building on the top of the slope to the building under the slope is used to reflect the influence of the elevation amplification effect of the seismic wave on the top building. The results show that the horizontal acceleration ratio between the top building and the under building is the largest at the height of 3–6 m, indicating that the buildings are most prone to damage at this height. This manuscript explains the research conclusion from the corresponding relationship between the natural vibration period of slope and the predominant frequency of seismic wave at the height of 3–6 m of top building.
4. The dynamic safety distance of building on the top of slope proposed in this manuscript is based on the physical and mechanical parameters of Q_3_ loess slope, the slope gradient range is from 25°to 70° and the height range is from 15 to 30 m. For buildings on the top of slope, it is applicable to buildings with no more than 8 floors. Since the seismic intensity input in the calculation is 0.12 g, the dynamic safety distance of the building on the top of slope proposed in this manuscript is applicable to the loess area of China with the basic fortification intensity of 7 degrees. The sliding range of the slope, the amplification and attenuation rules of seismic waves and the permanent displacement of the building under the action of higher intensity earthquakes need to be further discussed and analyzed.
